# Mitochondrial DNA Haplogroup JT is Related to Impaired Glycaemic Control and Renal Function in Type 2 Diabetic Patients

**DOI:** 10.3390/jcm7080220

**Published:** 2018-08-16

**Authors:** Noelia Diaz-Morales, Sandra Lopez-Domenech, Francesca Iannantuoni, Ester Lopez-Gallardo, Eva Sola, Carlos Morillas, Milagros Rocha, Eduardo Ruiz-Pesini, Victor M. Victor

**Affiliations:** 1 Service of Endocrinology, University Hospital Doctor Peset, Foundation for the Promotion of Health and Biomedical Research in the Valencian Region (FISABIO), 46017 Valencia, Spain; nodiazmo@alumni.uv.es (N.D.-M.); Sandra.lopez@uv.es (S.L.-D.); franian@alumni.uv.es (F.I.); eva.sola@uv.es (E.S.); carlos.morillas@uv.es (C.M.); 2 Department of Biochemistry and Molecular and Cell Biology, University of Zaragoza, 50013 Zaragoza, Spain; esterlop@unizar.es (E.L.-G.); eduruiz@unizar.es (E.R.-P.); 3 Instituto de Investigación Sanitaria de Aragón (IIS Aragón), 50013 Zaragoza, Spain; 4 Centro de Investigaciones Biomédicas En Red de Enfermedades Raras (CIBERER), 50013 Zaragoza, Spain; 5 CIBERehd-Department of Pharmacology and Physiology, University of Valencia, 46010 Valencia, Spain; 6 Fundación ARAID, 50018 Zaragoza, Spain

**Keywords:** type 2 diabetes mellitus, mitochondrial haplogroup, mtDNA, nephropathy, glycemic control

## Abstract

The association between mitochondrial DNA (mtDNA) haplogroup and risk of type 2 diabetes (T2D) is undetermined and controversial. This study aims to evaluate the impact of the main mtDNA haplogroups on glycaemic control and renal function in a Spanish population of 303 T2D patients and 153 healthy controls. Anthropometrical and metabolic parameters were assessed and mtDNA haplogroup was determined in each individual. Distribution of the different haplogroups was similar in diabetic and healthy populations and, as expected, T2D patients showed poorer glycaemic control and renal function than controls. T2D patients belonging to the JT haplogroup (polymorphism m.4216T>C) displayed statistically significant higher levels of fasting glucose and HbA_1c_ than those of the other haplogroups, suggesting a poorer glycaemic control. Furthermore, diabetic patients with the JT haplogroup showed a worse kidney function than those with other haplogroups, evident by higher levels of serum creatinine, lower estimated glomerular filtration rate (eGFR), and slightly higher (although not statistically significant) urinary albumin-to-creatinine ratio. Our results suggest that JT haplogroup (in particular, change at position 4216 of the mtDNA) is associated with poorer glycaemic control in T2D, which can trigger the development of diabetic nephropathy.

## 1. Introduction

Type 2 diabetes (T2D) has become one of the most common metabolic diseases, with a rapid increase in its prevalence over recent decades, representing an enormous cost to public health organisms. It is obvious that environmental factors—such as diet and physical activity—play a key role in the pathogenesis of T2D, but an emerging body of evidence suggests that genetic factors can play an important role in the development and severity of T2D [1,2]. Therefore, characterization of new parameters that allow us to identify individuals at a high risk of developing T2D or to predict a poor prognostic of the disease are likely to be of great use in clinical practice, for designing strategies for primary prevention and for personalising treatments according to each specific condition.

Mitochondrial dysfunction is well known to be involved in the pathophysiology of T2D, as it affects not only insulin secretion but also insulin resistance [3,4]. In this sense, genetic factors such as mitochondrial DNA (mtDNA) variations may affect mitochondrial function and lead to the development of diabetes [5]. For example, an mtDNA mutation at nucleotide m.3243A>G has been described to cause maternally inherited diabetes and deafness [6,7]. Another relatively common variant, mtDNA m.16189T>C, has been associated with an enhanced risk of type 2 diabetes in Asian [8] and European [9] populations. Mitochondrial DNA haplogroups are defined by common variants of single nucleotide point (SNP) mutations of the mtDNA that result in a division of the population into discrete groups, each of which shares a common maternal ancestor. Although some studies have suggested mitochondrial haplogroups are involved in the genetic susceptibility of T2D [10,11,12], this connection is not altogether clear, as other authors have reported that haplogroups are unlikely to play a role in the risk of developing this disorder [13,14]. 

Type 2 diabetes is characterised by inadequate metabolic control associated with subsequent micro- and macro-vascular complications. Fasting plasma glucose levels indicate how efficiently glucose levels are managed in the absence of dietary glucose, while glycated haemoglobin (HbA_1c_) provides information regarding average blood glucose levels over the previous 8-12 weeks, thus representing an objective measurement of glycaemic control [15]. An association between poor glycaemic control and enhanced risk of microvascular complications, such as nephropathy, has been widely reported in diabetic patients [16,17]. Furthermore, some authors have suggested an effect of mtDNA haplogroups on the risk of developing diabetic complications in T2D [11,18,19]. However, whether or not mitochondrial haplogroups are involved in the glycaemic control of type 2 diabetic patients and the subsequent development of microvascular complications, such as nephropathy, has not yet been studied. 

In the present study, we assessed a Spanish population of 303 T2D patients and 153 healthy controls with the aim of investigating differences in metabolic parameters and renal dysfunction markers according to the main mitochondrial macro-haplogroups.

## 2. Experimental Section

### 2.1. Subjects

Our study population was composed of 303 T2D patients and 153 healthy volunteers recruited at the Endocrinology and Nutrition Service of the University Hospital Dr. Peset (Valencia, Spain). T2D was diagnosed according to the criteria of the American Diabetes Association 2017 [20] (fasting plasma glucose ≥126 mg/dL, or 2-h plasma glucose ≥200 mg/dL after a 75 g oral glucose tolerance test, or HbA_1C_ ≥6.5%, or random plasma glucose ≥200 mg/dL). Subjects who met any of the following criterion were excluded from the study: history of cardiovascular disease (stroke, ischemic heart disease, peripheral vascular disease, and chronic disease related to cardiovascular risk); severe disease including malignances, autoimmune, inflammatory or infectious diseases; and abnormal haematological profile. 

Written informed consent was obtained from all the participants before they participated in the study. The study was conducted in accordance with the Helsinki Declaration, and approved by the Ethics Committee of the University Hospital Dr. Peset (Project identification code: 97/16).

### 2.2. Anthropometric and Biochemical Parameters

During the medical appointment, weight (kg), height (m), systolic and diastolic blood pressure (SBP, DBP; mm Hg), and waist and hip circumference (cm) were measured in all the participants. Body mass index (BMI; kg/m^2^) and waist-to-hip ratio (WHR) were then calculated.

Venous blood samples were collected in fasting conditions from both control and type 2 diabetic subjects and centrifuged at 1500 × g for 10 min at 4 °C to obtain serum, in which levels of glucose, total cholesterol and triglycerides were determined by means of an automated enzymatic method using a Beckman Synchron LX20 Pro analyzer (Beckman Coulter, Brea, CA, USA). High-density lipoprotein cholesterol (HDL-c) levels were measured using a direct method with a Beckman Synchron LX20 Pro analyzer (Beckman Coulter, Brea, CA, USA), and low-density lipoprotein cholesterol (LDL-c) was calculated with Friedewald’s formula. Insulin was measured with an Immulite 1000 automated immunoassay system (Siemens Healthcare SL, Madrid, Spain) and the homeostasis model assessment index of insulin resistance (HOMA-IR) was calculated to estimate insulin resistance using fasting insulin and glucose levels: HOMA = [fasting insulin (μU/mL) × fasting glucose (mg/dL)]/405. HOMA index was only calculated for patients not undergoing insulin therapy. Percentage of HbA_1c_ was measured by means of an automatic glycohemoglobin analyzer (Arkray Inc., Kyoto, Japan) and high-sensitive C-reactive protein (hs-CRP) levels were assessed with the Dade Behring Nephelometer II Analyzer System using an immunonephelometric assay (Dade Behring, Deerfield, IL, USA). 

Creatinine in serum and urine was determined by Jaffe’s reaction. Measurements of urinary albumin concentrations were performed by turbidimetry with an Architect c-16000 autoanalyzer (Abbott, Lake Bluff, IL, USA). Estimated glomerular filtration rate (eGFR) was calculated by the CKD-EPI equation from serum creatinine [21].

### 2.3. Haplotyping

Total DNA was extracted from whole blood with the REALPURE “SSS” Kit (Durviz SL, Valencia, Spain) and stored at −20 °C until analysis.

Mitochondrial haplogroups HV, JT and U were defined by the mtDNA polymorphisms m.7028C>T, m.12308A>G, m.4216T>C and m.14766T>C [22]. These haplogroups encompass around 90% of the Spanish population [23]. Samples revealing other haplogroups with low frequencies among the population (those not classified as HV, JT or U) were grouped altogether and referred to as “Others”. Custom designed Taqman® SNP genotyping assays (Applied Biosystems, Foster City, CA, USA) were used to analyse mtDNA genetic variants and samples were run in a Step One Plus Real Time PCR System (Applied Biosystems, Foster City, CA, USA). The analysis consisted of a pre-read and post-read step of the plate of 30 s at 60 °C, before and after the PCR cycle. The cycle conditions were 10 min at 95 °C, followed by 40 cycles of 15 s at 95 °C and 1 min at 60 °C. Information on the haplogroups, dyes, probes and primers in each assay is widely explained in Nogales-Gadea et al. [24]. For each genotype analysis, positive and negative controls from different previously characterised mtDNA aliquots were used to ensure an adequate internal control.

### 2.4. Statistical Analysis

Results were processed using SPSS Software version 17.0 (SPSS Statistics Inc., Chicago, IL, USA) for statistical analysis. Data in tables are presented as means ± standard deviation for normally distributed data, medians (25th and 75th quartile) for non-normally distributed data, or percentage for qualitative variables. Figures show mean and standard error of the mean. Potential differences between haplogroups were analysed by ANOVA for normally distributed variables and the Kruskal-Wallis test for non-normally distributed variables. When differences among groups were detected, Student-Newman-Keuls or Dunn’s multiple-comparison post hoc test were applied, as appropriate. Frequencies in T2D patients and control subjects were compared using the chi-square test. A Student’s *t*-test was employed to evaluate differences between controls and type 2 diabetic patients. The effect of possible covariates (such as age, sex, BMI or duration of diabetes) was analyzed with a univariate general linear model. For all the tests, a two-tailed *p* < 0.05 was considered significant. 

## 3. Results

### 3.1. Clinical Characteristics of the Study Population

Our observational study included 303 type 2 diabetic patients and 153 healthy controls. Haplogroup distribution, and anthropometrical and inflammatory characteristics, as well as lipid profile of the studied participants are shown in Table 1. 

Our cohort of healthy controls showed a haplogroup distribution similar to that reported in a larger Spanish population by Dahmany et al. [23]. No differences were found in the distribution of the different macro-haplogroups between control subjects and diabetic patients (*p* = 0.68). Although our cohort of T2D patients was characterised by higher age and percentage of men with respect to the control population (*p* < 0.001), when sub-classified by haplogroup, no differences were found in these parameters among haplogroups in the diabetic population (*p* = 0.92 for age and *p* = 0.22 for male percentage) and control subjects (*p* = 0.68 for age and *p* = 0.36 for male percentage). As expected, T2D patients in total had higher body mass index (BMI), waist-to-hip ratio (WHR), systolic blood pressure (SBP), diastolic blood pressure (DBP), high-sensitive C-reactive protein (hs-CRP) (*p* < 0.001) than control subjects. Lipid profile in the diabetic patients showed typical characteristics of atherogenic dyslipidemia, with elevated levels of triglycerides (*p* < 0.001 vs. control) and low levels of HDL-c (*p* < 0.001 when compared to control subjects). The lower levels of total cholesterol (*p* = 0.001) and LDL-c (*p* = 0.002) found in diabetic patients vs. controls were probably due to the fact that most of the patients were being treated with antihyperlipidemic agents (Table 2). No statistically significant differences in the studied parameters were detected according to haplogroup in the type 2 diabetic population or the control group (see Table 1 for *p*-values).

Pharmacologic treatment of the type 2 diabetic patients included in this study is shown in Table 2. No significant differences were observed between the percentages of patients treated with hypolipidemic, antidiabetic, and antihypertensive agents (see Table 2 for *p*-values).

### 3.2. Glucose Metabolism

**Figure 1:**
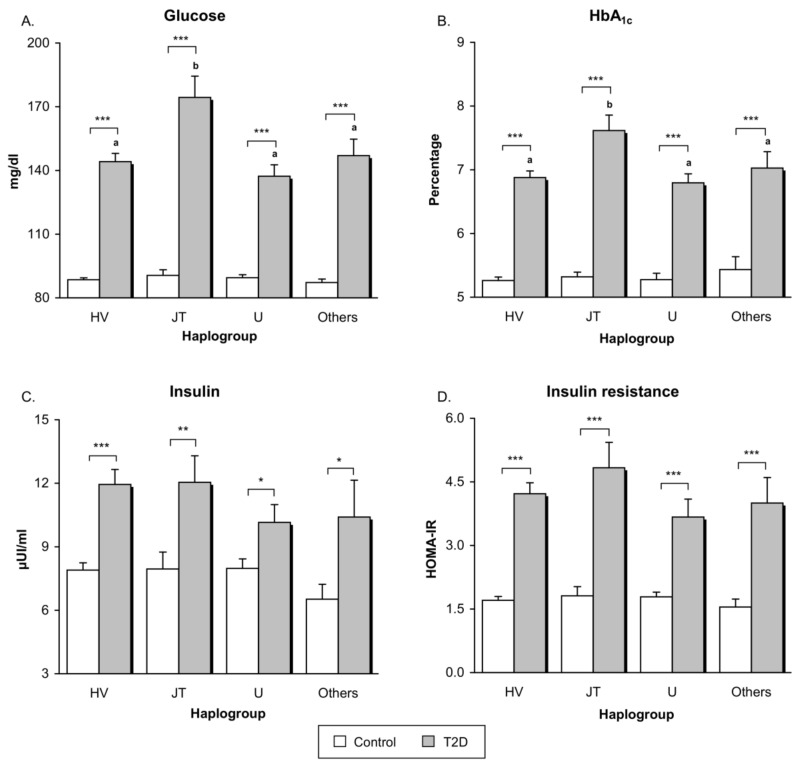
Glycaemic control and insulin resistance-related parameters in healthy controls and type 2 diabetic patients belonging to the main mitochondrial macro-haplogroups. (**A**) Serum levels of fasting glucose (expressed as mg/dL); (**B**) Percentage of glycated haemoglobin (HbA1c) levels; (**C**) Serum levels of fasting insulin (μUI/mL); (**D**) HOMA-IR index calculated as [fasting insulin (μU/mL) × fasting glucose (mg/dL)]/405. Only patients not treated with insulin therapy are included in the graphs of fasting insulin and HOMA-IR. Grey bars show type 2 diabetic patients and white bars represent controls. * *p* < 0.05; ** *p* < 0.01; *** *p* < 0.001 when compared controls vs. type 2 diabetic subjects. Letters indicate significant differences among type 2 diabetic patients with different haplogroups (*p* < 0.05) when compared by means of one-way ANOVA followed by Student-Newman-Keuls post-hoc test (i.e., bars tagged with the same letter do not differ significantly from each other, while bars with no letter in common are significantly different from each other (*p* < 0.05)). Abbreviations: HbA1c, glycated haemoglobin; HOMA-IR, Homeostasis model assessment index of insulin resistance; T2D, Type 2 diabetes.

First analysis performed was a comparison between T2D patients and controls as a whole, without subdividing by haplogroup (Appendix Table A1). As expected, type 2 diabetic patients showed higher levels of fasting glucose, HbA_1c_, fasting insulin and HOMA-IR index than control subjects (*p* < 0.001). Differences between controls and subjects with T2D remained statistically significant after adjustment for age, sex, and BMI (*p* < 0.001 for glucose, HbA_1c_ and HOMA; *p* = 0.01 for insulin). Graphs showing parameters related with glycaemic control and insulin resistance are plotted in Figure 1. Differences in glucose, HbA1c, insulin, and HOMA between control subjects and T2D patients remained significant after subdivided by haplogroup (*p* < 0.001 for glucose, HbA1c, and HOMA when comparing control vs T2D belonging to haplogroups HV, JT, U, and Others. For insulin levels: *p* < 0.001 when comparing control vs T2D in haplogroup HV; *p* < 0.01 in haplogroup JT; and *p* < 0.05 in haplogroups U and Others. Differences in *p*-values found in the levels of insulin are attributable to differences in the sample size between haplogroups).

Interestingly, diabetic patients with the JT haplogroup showed significantly higher levels of fasting glucose (*p* = 0.001) and HbA_1c_ (*p* = 0.007) compared to patients belonging to the other haplogroups analysed (grey bars in Figure 1A,B). These differences remained statistically significant despite adjustments for duration of diabetes (*p* = 0.006 for fasting glucose and *p* = 0.002 for HbA_1c_). Nevertheless, no differences were found in the levels of fasting insulin (*p* = 0.50) and HOMA-IR index (*p* = 0.38) when T2D patients with different haplogroups were compared (grey bars in Figure 1C,D). Control subjects did not reveal differences depending on haplogroup for any of the parameters related with glycaemic control and insulin resistance (white bars in Figure 1; glucose: *p* = 0.70; HbA_1c_: *p* = 0.81; insulin: *p* = 0.60; HOMA-IR: *p* = 0.83).

### 3.3. Renal Function

Type 2 diabetic patients, when analyzed as a whole, exhibited lower kidney function than control subjects, expressed by higher serum creatinine concentrations (*p* < 0.001) and lower eGFR (*p* < 0.001) (Appendix Table A1). Differences in eGFR between controls and patients remained when adjusted by age, sex, and BMI (*p* = 0.04), whereas differences in creatinine levels were not longer statistically significant after adjusting for age, sex, and BMI (*p* = 0.12). After subdivided by haplogroup, differences between T2D patients and controls remained statistically significant only in haplogroups HV (*p* < 0.001) and JT *p* < 0.05 for serum creatinine, and in haplogroups HV (*p* < 0.01), JT (*p* < 0.05), and Others (*p* < 0.05) for eGFR. Differences in *p*-values found between the different haplogroups are probably due to differences in the sample size.

**Figure 2:**
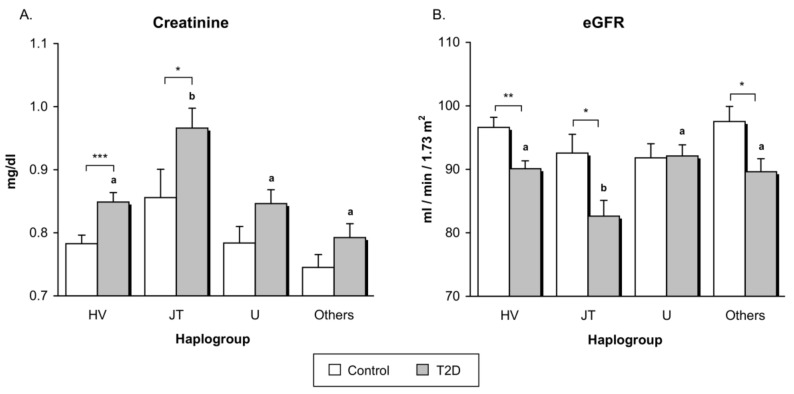
Assessment of renal function in healthy volunteers and type 2 diabetic patients subdivided by mitochondrial haplogroup. (**A**) Concentrations of serum creatinine; (**B**) Levels of estimated glomerular filtration rate (eGFR). White bars correspond to controls, while grey bars represent type 2 diabetic patients. * *p* < 0.05; ** *p* < 0.01; *** *p* < 0.001 in controls vs. type 2 diabetic subjects. Letters indicate significant differences among type 2 diabetic patients with different haplogroups (*p* < 0.05) when compared by means of one-way ANOVA followed by Student-Newman-Keuls post-hoc test (i.e., bars tagged with the same letter do not differ significantly from each other, while bars with no letter in common are significantly different from each other, (*p* < 0.05)). Abbreviations: eGFR, estimated glomerular filtration rate; T2D, Type 2 diabetes.

In the case of T2D patients, those with the JT haplogroup showed a worse renal function than patients belonging to HV, U, and Others haplogroups, manifested as significantly higher levels of serum creatinine (grey bars in Figure 2A, *p* < 0.001) and lower eGFR (grey bars in Figure 2B, *p* = 0.01). Differences between the JT group and all the other macro-haplogroups in creatinine levels and eGFR did not change in the diabetic population after adjusting by duration of diabetes (*p* < 0.001 for creatinine levels and *p* = 0.003 for eGFR). Control subjects did not reveal statistically significant differences in kidney function according to haplogroups (white bars in Figure 2; *p* = 0.09 for creatinine and *p* = 0.27 for eGRF).

In light of the above mentioned results, we also analyzed concentrations of urinary albumin and creatinine in patients in whom said parameters were measured on the same day as blood was extracted; namely, in a total of 106 type 2 diabetic patients (52 from the HV group, 16 from the JT group, 26 from the U group, and 12 from the “Others” group). Urinary albumin-to-creatinine ratio (mg/g) was slightly higher in the JT group (19.46 ± 17.54 mg/g) than in those belonging to the other haplogroups (HV: 12.55 ± 7.92 mg/g; U: 12.54 ± 8.73 mg/g; others: 12.49 ± 7.33 mg/g), although it did not reach statistical significance in the one-way ANOVA test (*p* = 0.099).

## 4. Discussion

In the present study, we have performed a case-control study to explore the possible effects of the main mitochondrial haplogroups on metabolic control and renal function in a Spanish population of 303 type 2 diabetic patients and 153 healthy controls. We have observed that T2D patients belonging to the JT macrohaplogroup showed enhanced levels of fasting plasma glucose, HbA1c, creatinine, and decreased eGFR when compared to patients from the other haplogroups (HV, U, and Others), thus suggesting poorer metabolic control and renal function in T2D patients with the JT haplogroup.

Mitochondria are responsible for the cell’s energy supply through oxidative phosphorylation (OXPHOS), and some of the proteins involved in this process are encoded in the mtDNA. Given the importance of OXPHOS in insulin secretion [25,26], different genetic variants are potential candidates for playing a role in the susceptibility to or protection against metabolic defects [13]. Mitochondrial haplogroups are clusters of phylogenetically related mtDNA haplotypes that might have been selected during evolution to permit humans to adapt to famine or cold climates [27]. It has been suggested that these mtDNA variants contribute to energy metabolism and, hence, may be associated with metabolic diseases [28]. Crispim et al. [10] reported that the European-specific JT mitochondrial haplogroup was associated with insulin resistance and type 2 diabetes in Caucasian-Brazilian patients, as patients belonging to the JT cluster exhibited higher levels of HOMA-IR. In addition, the J1 haplogroup is thought to be involved in susceptibility to type 2 diabetes among Caucasian (Jewish) patients depending on family health history [29]. According to several studies performed in Asian populations, individuals carrying haplogroup N9a are less susceptible to type 2 diabetes and metabolic syndrome [30,31]. However, in spite of this evidence, the association between mitochondrial haplogroups and type 2 diabetes is not clear, with many studies providing conflicting results or failing to find significant associations [13,14,18,32]. Our results do not show a direct association of the development of T2D with the main macro-haplogroups, as no differences were found in the frequencies of each haplogroup between our diabetic and control populations. Interestingly, we found that patients belonging to the JT cluster presented poorer glycaemic control and higher levels of fasting glucose and HbA1c, than other patients, thus suggesting that said haplogroup is involved in the metabolism of glucose in patients with T2D. Our findings are in agreement with those reported by Crispim et al. [10] which described higher levels of HOMA-IR in patients with JT haplogroup, although no statistically significant differences were found in our cohort of type 2 diabetic patients, probably because the size of our sample was smaller than the sample size in the cited work.

Type 2 diabetes and inadequate glycaemic control are frequently associated with macro- and micro-vascular complications. Whether or not mitochondrial haplogroups play a role in modulating the development of T2D-related complications is a question that has been widely studied. Achilli et al. [18] found an association of various mitochondrial haplogroups and increased risk of diabetic complications in an Italian population: haplogroup H3 increased the probability of developing neuropathy; haplogroup H was linked to retinopathy; and subjects harbouring V and U3 mtDNA showed enhanced incidence of renal failure and nephropathy. In this context, it is worth pointing out that diabetic nephropathy has been associated with specific mitochondrial haplogroups in several studies; for instance, Feder et al. [33] reported a link with the J1 haplogroup in an Ashkenazi Jewish population, while Niu et al. [11] reported a link with the N9a haplogroup in a Chinese population. Our data are in accordance with an involvement of mitochondrial haplogroup in the development of nephropathy, as our type 2 diabetic patients belonging to the JT haplogroup showed higher levels of serum creatinine and lower levels of eGFR compared to patients belonging to the other haplogroups analyzed. Interestingly, though not statistically significant, T2D patients harbouring the JT haplogroup also presented a higher urinary albumin-to-creatinine ratio. Taken together, these results suggest T2D patients with the JT haplogroup are likely to have impaired kidney function. 

The variant m.4216T>C, a key SNP for defining the JT macro-haplogroup [34], leads to a non-synonymous amino acid change in the mtDNA *MT-ND1* gene encoding NADH:Ubiquinone oxidoreductase core subunit 1 (p.MT-ND1), one of the components of the mitochondrial respiratory complex I. Electrons coming from glucose metabolism through glycolysis and the Krebs cycle are principally stored in NADH for ATP production and oxygen reduction. It has been proposed that hyperglycaemia can increase the production of the complex I substrate NADH [35]. Overproduction of NADH leads to an electron pressure on the mitochondrial electron transport chain that drives to an increase in electron leakage and the subsequent high production of reactive oxygen species (ROS) [36,37]. As the major enzyme implicated in NADH recycling, mitochondrial complex I impairment can lead to further increased levels of NADH [38], with the following enhancement of ROS. Altogether these mechanisms will induce oxidative stress, which has been widely reported as a central player in the development of insulin resistance, pancreatic β-cells dysfunction, and finally, type 2 diabetes [39,40,41]. A recent study has demonstrated that pharmacological inhibition of complex I of mitochondrial electron transport chain improves glucose homeostasis and ameliorates hyperglycaemia [42]. Our findings bring us to hypothesize that variant m.4216T>C results in aberrant activity of complex I that, under diabetic conditions, leads to poorer glycaemic control. For this reason, we suggest that inhibitors of complex I—such as metformin and thiazolidinediones [43]—may be adequate drugs for the treatment of T2D patients belonging to the JT haplogroup.

Both hyperglycaemia and excessive oxidative stress are well known to be involved in the development of diabetic vascular complications [44,45], including microvascular complications such as nephropathy [46]. The kidney is especially vulnerable to the damage produced by hyperglycaemia-induced oxidative stress; in fact said damage has been suggested as an important mechanism involved in the pathogenesis of tubular and glomerular abnormalities [46]. 

The present study has some limitations in terms of statistical power. We did not perform a previous sample size estimation due to the lack of studies addressing the role of the haplogroups here studied (HV, JT, U, and others) on metabolic control and renal function. However, we consider that our study has enough power for reaching statistically significant differences among different haplogroups in the diabetic population. Nevertheless, researches in larger populations will serve to confirm these results.

Together, this evidence leads us to hypothesize that the JT haplogroup (in particular, change at position 4216 of the mtDNA) might result in a poorer glycaemic control in type 2 diabetic patients, thus contributing to the development of diabetic nephropathy. Future studies with a larger sample size would help to confirm our results and, if corroborated, haplogroup screening in recently diagnosed T2D patients might be suggested as a way of predicting disease progression and choosing the most adequate clinical treatment for avoiding macro and microvascular-associated complications.

## Figures and Tables

**Table 1:** Haplogroup distribution and characteristics of the study population. Anthropometrical, inflammatory and lipid profile.

	Healthy Controls						Type 2 Diabetic Patients						*p*-value *
Haplogroup	HV	JT	U	Others	Total	*p*-value	HV	JT	U	Others	Total	*p*-value	
N (%)	81 (52.9)	26 (17.0)	35 (22.9)	11 (7.2)	153 (100)	-	165 (54.5)	43 (14.2)	65 (21.4)	30 (9.9)	303 (100)	-	0.68
Male %	37.0	41.9	23.7	27.3	34.2	0.36	68.5	81.8	77.3	66.7	72.1	0.22	<0.001
Age (years)	41.3 ± 16.4	45.3 ± 16.0	41.5 ± 15.7	40.0 ± 14.0	41.9 ±16.0	0.68	58.6 ± 9.8	59.5 ± 9.3	58.4 ± 9.9	59.3 ±7.8	58.8 ± 9.5	0.92	<0.001
BMI (kg/m^2^)	24.4 ± 3.7	25.6 ± 4.5	25.2 ± 4.2	23.7 ± 2.3	24.8 ± 24.8	0.39	30.8 ± 4.8	30.2 ± 3.8	30.1 ± 4.4	31.2 ± 4.2	30.6 ± 4.5	0.68	<0.001
WHR	0.83 ± 0.08	0.84 ± 0.13	0.85 ± 0.09	0.79 ± 0.08	0.83 ± 0.09	0.41	0.98 ± 0.08	0.97 ± 0.06	1.00 ± 0.08	0.98 ± 0.07	0.98 ± 0.08	0.58	<0.001
SBP (mmHg)	122 ± 18	123 ± 21	122 ± 18	116 ± 16	122 ± 18	0.80	133 ± 18	132 ± 15	135 ± 18	137 ± 18	134 ± 17	0.79	<0.001
DBP (mmHg)	73 ± 10	73 ± 10	77 ± 14	72 ± 11	74 ± 11	0.45	78 ± 10	79 ± 11	79 ± 9	77 ± 11	78 ± 10	0.88	<0.001
Duration of diabetes	-	-	-	-	-	-	10.1 ± 8.4	9.7 ± 6.7	11.0 ± 8.5	10.6 ± 6.7	10.2 ± 8.0	0.87	-
Hs-CRP (mg/L)	0.82 (0.45–2.23)	1.07 (0.40–3.16)	1.04 (0.45–2.86)	1.24 (0.31–2.60)	1.01 (0.45–2.60)	0.76	2.57 (1.19–5.76)	2.34 (1.09–5.24)	2.56 (0.95–5.61)	2.53 (1.04–5.20)	2.53 (1.08–5.50)	0.86	<0.001
TC (mg/dL)	189 ± 35	194 ± 29	190 ± 42	203 ± 32	191 ± 36	0.62	180 ± 41	181 ± 45	170 ± 36	182 ± 33	178 ± 40	0.35	0.001
HDL-c (mg/dL)	55.9 ± 16.5	54.5 ± 16.0	54.7 ± 11.6	59.9 ± 13.1	55.7 ± 15.1	0.76	42.8 ± 11.0	41.9 ± 11.4	41.4 ± 9.5	44.3 ± 9.5	42.5 ± 10.6	0.62	<0.001
LDL-c (mg/dL)	115 ± 31	122 ± 28	118 ± 35	125 ± 24.9	118 ± 30.9	0.67	109 ± 36.0	108 ± 37.3	100 ± 33.1	109 ± 32.4	107 ± 35.3	0.34	0.002
Triglycerides (mg/dL)	69 (51–114)	70 (47–104)	75 (58–94)	86 (60–117)	73 (54–104)	0.63	122 (88–176)	123 (101–156)	129 (94–200)	134 (101–172)	125 (93–174)	0.94	<0.001

Normally distributed data are shown as mean ± SD and non-normally distributed data as median (25th–75th quartiles). *p*-value * when comparing type 2 diabetic patients (Total) vs. healthy controls (Total). Abbreviations: BMI, body–mass index; DBP, diastolic blood pressure; HDL-c, high-density lipoprotein cholesterol; hs-CRP, high-sensitive C-reactive protein; LDL-c, low-density lipoprotein cholesterol; SBP, systolic blood pressure; SD, standard deviation; TC, total cholesterol; WHR, waist-to-hip ratio.

**Table 2:** Pharmacological treatment of the study’s cohort of type 2 diabetic patients.

	HV	JT	U	Others	Total	*p*-value
Statins (%)	56.5	50.0	53.2	56.0	54.7	0.91
Fibrate (%)	13.0	19.4	8.5	12.0	13.0	0.54
Ezetimibe (%)	3.2	5.0	6.7	0.0	4.0	0.73
Metformin (%)	63.8	83.3	77.6	60.0	69.5	0.06
DPP-4 inhibitors (%)	33.9	45.0	41.9	42.9	38.6	0.76
Insulin (%)	29.3	19.4	30.6	20.0	27.0	0.51
Sulfonilureas (%)	18.8	14.3	10.4	33.3	17.8	0.11
Glitazones (%)	7.8	19.4	4.1	12.0	9.3	0.09
Glinides (%)	21.6	16.7	8.2	32.0	19.0	0.07
GLP-1 agonists (%)	19.4	15.0	6.5	21.4	15.7	0.40
Antihypertensive (%)	45.2	43.2	49.0	48.0	46.0	0.95

Abbreviations: DPP-4, dipeptidil peptidasa-4; GLP-1, glucagon-like peptide-1.

## Appendix

**Table A1:** Differences in glycaemic control and renal function between controls and type 2 diabetic patients.

	Healthy Controls	Type 2 Diabetic Patients	*p*-value	Age, Sex, and BMI Adjusted *p*-value
Glucose (mg/dL)	89.04 ± 8.93	147.39 ± 50.87	<0.001	<0.001
HbA_1c_ (%)	5.28 ± 0.32	6.99 ± 1.32	<0.001	<0.001
Insulin (μUI/mL)	7.84 ± 2.98	11.50 ± 5.91	<0.001	0.013
HOMA-IR	1.73 ± 0.76	4.21 ± 2.39	<0.001	<0.001
Creatinine (mg/dL)	0.79 ± 0.12	0.86 ± 0.17	<0.001	0.12
eGFR (mL/min/1.73 m^2^)	94.92 ± 9.93	89.39 ± 13.19	<0.001	0.04

Data are shown as mean ± SD. Abbreviations: BMI, body–mass index; eGFR, estimated glomerular filtration rate; HbA1c, glycated haemoglobin; HOMA-IR: Homeostasis model assessment index of insulin resistance; SD: standard deviation.
